# The Melody Project: A Music-Based Intervention to Reduce Loneliness and Foster Belonging in Assisted Living

**DOI:** 10.1093/geroni/igaf122.3861

**Published:** 2025-12-31

**Authors:** Gemma Wang, Lena Thompson, Jordan Lewis

**Affiliations:** The Chapin School, New York, NY, New York, United States; University of Alaska Fairbanks, Fairbanks, Alaska, United States; University of Alaska Fairbanks, Fairbanks, Alaska, United States

## Abstract

Despite living in communal environments, approximately 55% of older adults in assisted living facilities experience loneliness, which is linked to a 29% higher risk of premature death. The Melody Project, developed by Gemma Wang, strives to address this issue. Moved by the isolation of assisted living residents living near her school, Wang drew on her eleven years of musical experience to lead music-based sessions at nearby facilities. After recognizing the residents’ profound joy, she established The Melody Project to continue fostering intergenerational engagement. This initiative hosts sessions connecting music and interaction through icebreaker activities, collaborative art inspired by music, and group discussions. The Melody Project has reached three nursing homes in New York City and has been received with positive feedback by residents and staff. The activities leave older adults with personalized keepsakes to remember their time with the students, and they remain deeply touched by the stories shared and relationships forged. With the support of her mentors, Wang plans to implement pre-post evaluation surveys to provide quantitative support for The Melody Project in the coming school year. She has also begun making connections in different states to establish state chapters and is researching aging and neurodegeneration at the University of California, Santa Barbara, this summer. By bridging generational divides through creative expression, The Melody Project reduces loneliness while fostering empathy, purpose, and belonging across generations, promoting meaningful relationships that help rediscover community and camaraderie. The Melody Project is thus a scalable, community-based, and evidence-informed approach to aging well together.

